# Prolonged Antigen Presentation Is Required for Optimal CD8+ T Cell Responses against Malaria Liver Stage Parasites

**DOI:** 10.1371/journal.ppat.1000877

**Published:** 2010-05-06

**Authors:** Ian A. Cockburn, Yun-Chi Chen, Michael G. Overstreet, Jason R. Lees, Nico van Rooijen, Donna L. Farber, Fidel Zavala

**Affiliations:** 1 Johns Hopkins Malaria Research Institute and Department of Molecular Microbiology and Immunology, Bloomberg School of Public Health, Baltimore, Maryland, United States of America; 2 Department of Surgery, University of Maryland at Baltimore, Baltimore, Maryland, United States of America; 3 Vrije Universiteit, VUMC, Department of Molecular Cell Biology, Faculty of Medicine, Amsterdam, The Netherlands; Case Western Reserve University, United States of America

## Abstract

Immunization with irradiated sporozoites is currently the most effective vaccination strategy against liver stages of malaria parasites, yet the mechanisms underpinning the success of this approach are unknown. Here we show that the complete development of protective CD8+ T cell responses requires prolonged antigen presentation. Using TCR transgenic cells specific for the malaria circumsporozoite protein, a leading vaccine candidate, we found that sporozoite antigen persists for over 8 weeks after immunization—a remarkable finding since irradiated sporozoites are incapable of replication and do not differentiate beyond early liver stages. Persisting antigen was detected in lymphoid organs and depends on the presence of CD11c+ cells. Prolonged antigen presentation enhanced the magnitude of the CD8+ T cell response in a number of ways. Firstly, reducing the time primed CD8+ T cells were exposed to antigen *in vivo* severely reduced the final size of the developing memory population. Secondly, fully developed memory cells expanded in previously immunized mice but not when transferred to naïve animals. Finally, persisting antigen was able to prime naïve cells, including recent thymic emigrants, to become functional effector cells capable of eliminating parasites in the liver. Together these data show that the optimal development of protective CD8+ T cell immunity against malaria liver stages is dependent upon the prolonged presentation of sporozoite-derived antigen.

## Author Summary

Malaria causes the deaths of 0.5–2 million people each year, mainly in Africa. A safe and effective vaccine is likely needed for the control or eradication of this disease. It has been shown that if people are bitten by malaria-infected mosquitoes that have been irradiated they are protected against fully infectious bites. Radiation makes the parasites in the mosquito (called sporozoites) incapable of dividing; however, the irradiated parasites are still capable of stimulating the immune system to provide protection against fully infectious parasites. Many trial vaccines are based on trying to simulate the immune response induced by irradiated sporozoites in a safe and cost-effective way. We want to know how sporozoites are able to efficiently induce immune responses. We found that sprozoite molecules are retained by the immune system for months after immunization. These molecules continue to stimulate the cells of the immune system such that they divide more and thus probably protect better. The ability of the immune system to retain parasite material long after the parasites themselves have disappeared is remarkable and gives new insights into how effective immune responses can be generated by vaccines.

## Introduction

Immunization with irradiated malaria sporozoites can induce sterile protection against subsequent challenge with live parasites in both mice and men [1], [2]. In murine models protection is mediated at least in part by CD8+ T cells [3], [4]. Understanding the mechanistic underpinnings of this protective response is considered essential if we are to emulate it with a practical and effective vaccine. It has been proposed previously, based on the detection of parasite remnants in the livers of immunized mice, that antigen persists after sporozoite immunization and that this may help generate protective immune responses [5]; however, there is no evidence that this material, assumed to be of parasite origin, is immunogenic or that it is presented to effector cells of the immune system.

The requirement for antigen in the full development of effector and memory responses in different microbial systems is an area of controversy. Studies, that are now considered seminal, have proposed that only a short (<24 hour) exposure to antigen is required to trigger the complete developmental program of CD8+ T cells [6], [7], [8]. Following initial antigen exposure CD8+ T cells expand into effector cells before forming a numerically stable memory population, a process that has been characterized as T cells on “autopilot” [9]. In the longer term, persisting antigen during chronic viral infection is often considered detrimental to T cell immunity as over-stimulated T cells may become exhausted and lose effector function [10], [11]. However, not all data suggests short exposures to antigen are optimal for T cell responses. The largest antigen specific CD8+ T cell responses observed in humans are seen during chronic infection with various Herpesviruses [12], [13]. For Herpesviruses and other chronic infections it maybe that continued recruitment of thymic emigrants by the continued presence of foreign antigen can maximize and maintain the immune response [14], [15]. Recently a number of authors have reported antigen persistence after acute viral infections [16], [17], [18]. Although the significance of this prolonged antigen presentation is not fully understood, some authors have suggested that it may have an important role in maintaining protective CD8+ T cell responses [18], while others suggest that this process contributes little to CD8+ T cell mediated immunity [19].

Most studies examining the effect of antigen exposure on CD8+ T cell responses have studied persistence following infection with live pathogens, often with little distinction being made between the duration of infection and the duration of antigen presentation. Irradiated *Plasmodium* sporozoites are incapable of division and differentiation and induce little or no inflammation [20] yet they stimulate robust protective CD8+ T cell responses; therefore, this is an excellent system to study whether antigen alone can persist in the absence of persisting infection. By transferring TCR transgenic T cells specific for the circumsporozoite (CS) protein, a leading vaccine candidate antigen, we were able to show that CS antigen persists for more than 8 weeks after immunization. Our studies show that prolonged antigen presentation drives the maximal expansion of effector cells, can stimulate the proliferation of established memory cells and recruit naïve cells, including recent thymic emigrants to the immune response. Thus prolonged antigen presentation may be required for the full development of CD8+ T cell mediated protective immunity.

## Results

### Prolonged antigen presentation after sporozoite immunization

**Figure 1:**
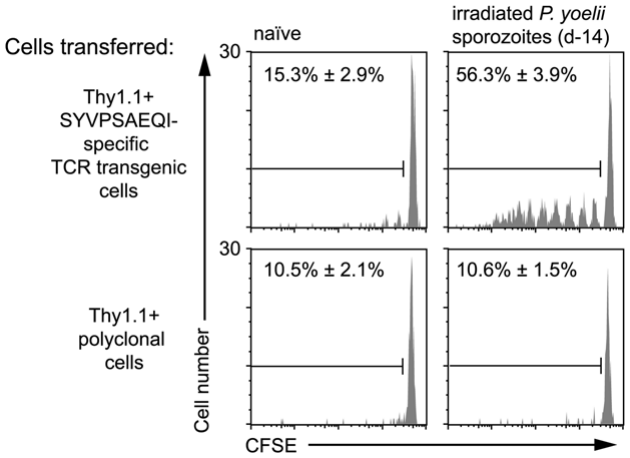
Prolonged antigen presentation after sporozoite immunization. 2×10^6^ Thy1.1+ CD8+ T cells from either TCR transgenic or WT animals were transferred into groups of naïve mice or mice immunized 2 weeks previously with 5×10^4^ irradiated *P. yoelii* sporozoites. 10 days after cell transfer the spleen cells from the recipient mice were analyzed by FACs. Histograms show CFSE profiles of the Thy1.1+ CD8+ cell populations in each group. Values are the mean ± SE of the percentage of divided cells (n = 3, data from one of two similar experiments shown).

To determine whether antigen persists following irradiated sporozoite immunization we transferred high numbers of CFSE labeled TCR transgenic Thy1.1+ CD8+ T cells specific for the CS protein of *P. yoelii* (hereafter referred to as “transgenic cells”) into mice that had been immunized 14 days previously with irradiated *P. yoelii* sporozoites or naïve control animals. We found that transgenic cells in previously immunized mice, but not naïve controls, had some dilution of the CFSE label showing that some of the cells had proliferated (Figure 1). T cell proliferation was not seen among polyclonal T cells from WT Thy1.1+ mice transferred to immune mice, indicating that proliferation was antigen specific and not due to bystander activation (Figure 1). Moreover, immunization with the related parasite *P. berghei* which express a different CD8+ epitope not recognized by our transgenic cells also failed to induce specific T cell proliferation further excluding the possibility of bystander activation (Figure S1A).

**Figure 2:**
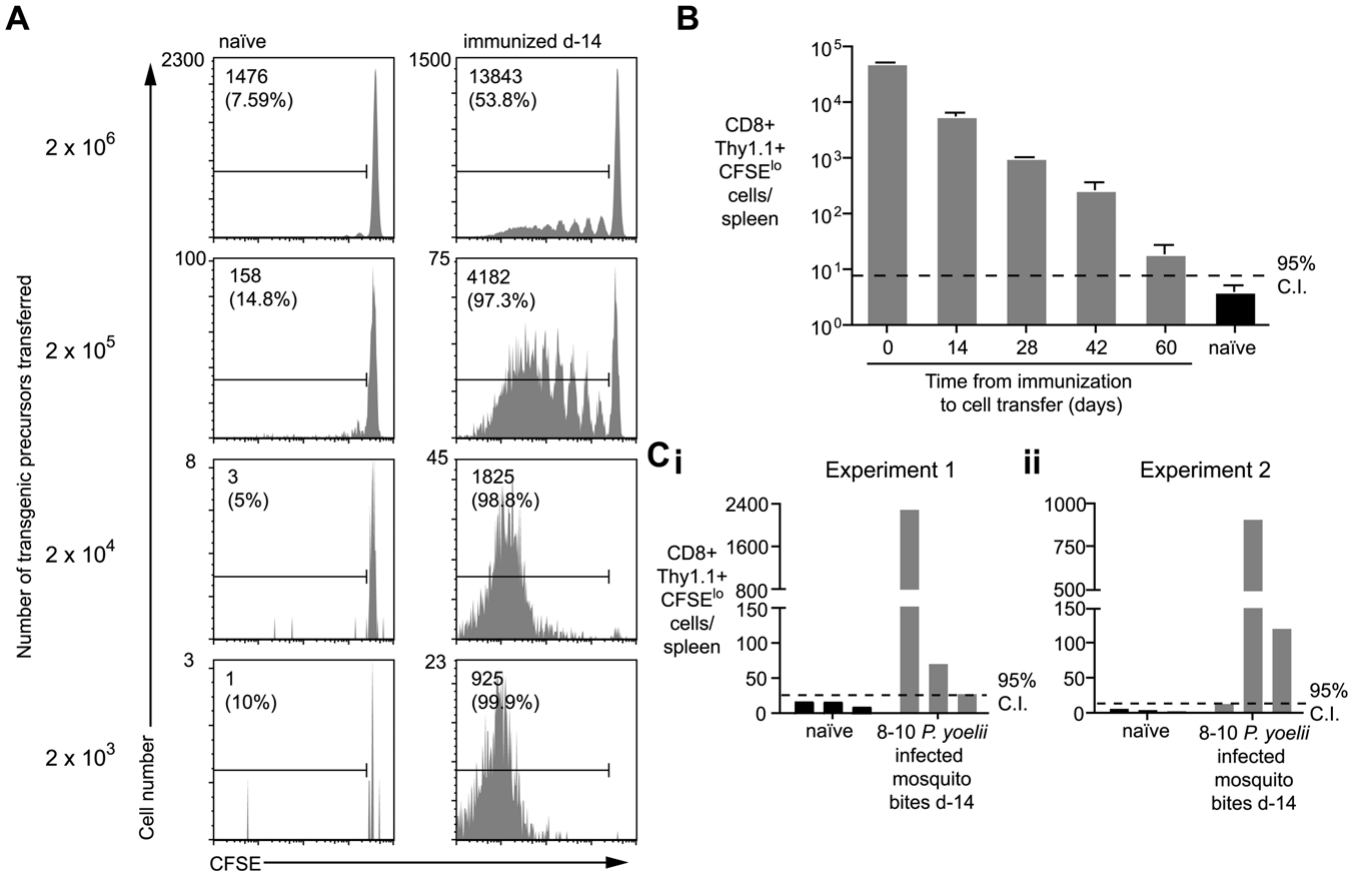
Duration of antigen presentation, and antigen persistence after mosquito biting. A. Mice were immunized with 5×10^4^ irradiated sporozoites i.v. and 14 days later 2×10^3^–2×10^6^ CFSE labeled transgenic cells were transferred to the mice and to unimmunized controls. Ten days later transgenic cells were enriched from the spleen and their CFSE profile determined by FACs. Representative plots from one of three mice per group are shown. Values given are the total number of divided transgenic (CD8+ Thy1.1+ CFSE^lo^) cells isolated from the spleen and in parentheses the % of total recovered transgenic cells that have divided (data representative of numerous similar experiments). B. Mice were immunized as in A. and 1×10^4^ transgenic cells were transferred to mice at different time points after immunization. Ten days after transfer the cells were recovered and the number of divided transgenic cells recovered was determined (n = 3, mean ± SE; data representative of two similar experiments). C. Three mice were bitten by 8–10 infected *P. yoelii* infected mosquitoes. Fourteen days later 1×10^4^ transgenic cells were transferred to the mice and 10 days later the number of divided transgenic cells was determined. Bars represent values for individual mice; data shown from two independent experiments (i and ii).

In the previous experiment, the proliferation of transgenic cells, transferred weeks after initial immunization, suggested the presence of persisting antigen. However, since T cell proliferation was limited we initially hypothesized that the amount of antigen that persists may be small and the phenomenon of marginal significance. Alternatively, we reasoned that the transfer of high numbers of transgenic cells might be inhibiting a more robust proliferative response that might be seen with more physiological numbers of cells [21]. Accordingly, we titrated different numbers of labeled transgenic cells into previously immunized mice and later isolated the transgenic cells from the spleen by magnetic bead separation to determine the degree of proliferation in the antigen specific cell population (Figure S2). Strikingly the extent of transgenic cell proliferation substantially increased if fewer precursor cells were used (Figure 2A): in mice that received 2×10^6^ cells only ∼50% of the recovered cells had proliferated - most of them fewer than 6 times; however, in mice that received more physiological numbers of transgenic cells essentially all the transgenic cells had divided and diluted out the CFSE label completely. Similarly to earlier experiments, *P. berghei* immunization did not induce transgenic cell proliferation even if low numbers of cells were transferred (Figure S1B). In subsequent experiments where we wished to analyze the differentiation of cells primed by persisting antigen, a low number of cells (2×10^3^) was transferred; where we were concerned with detecting only the presence or absence of persisting antigen higher numbers of cells were used (1×10^4^–2×10^5^) which removed the need for technically challenging T cell purification.

Using this sensitive *in-vivo* assay to measure continued antigen presentation, we were able to detect antigen in mice immunized up to 60d previously (Figure 2B). Importantly, we also found robust proliferation among transgenic cells transferred to mice that had been immunized 2 weeks previously via the bites of infected mosquitoes - the natural route - indicating that antigen persistence is a physiological phenomenon and not an artifact of needle injection of sporozoites (Figure 2C).

### Prolonged antigen presentation does not depend on the presence of parasite remnants in the liver

**Figure 3:**
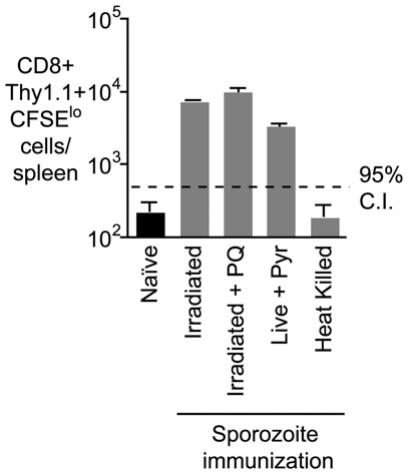
Prolonged antigen presentation does not depend on the presence of parasite. Mice were immunized i.v. with 5×10^4^ of each of irradiated sporozoites, irradiated sporozoites followed by 2 doses of PQ treatment 10 hours apart 7 days after immunization, live sporozoites (with pyrimethamine treatment to remove blood stage parasites) and heat killed sporozoites,. Fourteen days later 2×10^5^ transgenic cells were transferred to the immunized and naïve mice, 10 days after transfer the number of transgenic cells/ spleen was determined for each group of mice (n = 3, mean ± SE; data from one of three experiments).

It has been previously hypothesized that antigen persists in the liver as exo-erythrocytic remnants following irradiated sporozoite immunization [5]; in this study these remnants were not detected after live sporozoite infection, or in mice that were immunized with irradiated sporozoites and subsequently treated with primaquine (PQ) [5]. To determine if such exo-erythrocytic remnants could be the source of antigen that we detect, we immunized mice with irradiated sporozoites and treated with PQ 7 days later. The dose, route and timing of PQ treatment were as described in the original study [5]. Surprisingly, we found that PQ treatment did not have an effect on the presence of antigen after immunization (Figure 3). Moreover when we challenged with live parasites followed by pyrimethamine treatment we also detected persisting antigen (Figure 3). Nonetheless we determined that metabolically active parasites (live or irradiated) are required for the establishment of antigen persistence as antigen was not detected after immunization with heat-killed sporozoites (Figure 3). Together these data show that while metabolically active live or attenuated parasites are required to establish antigen persistence, this persistence does not require continued parasitic infection.

### Antigen persists in the lymph node draining the site of immunization, the spleen and the liver

**Figure 4:**
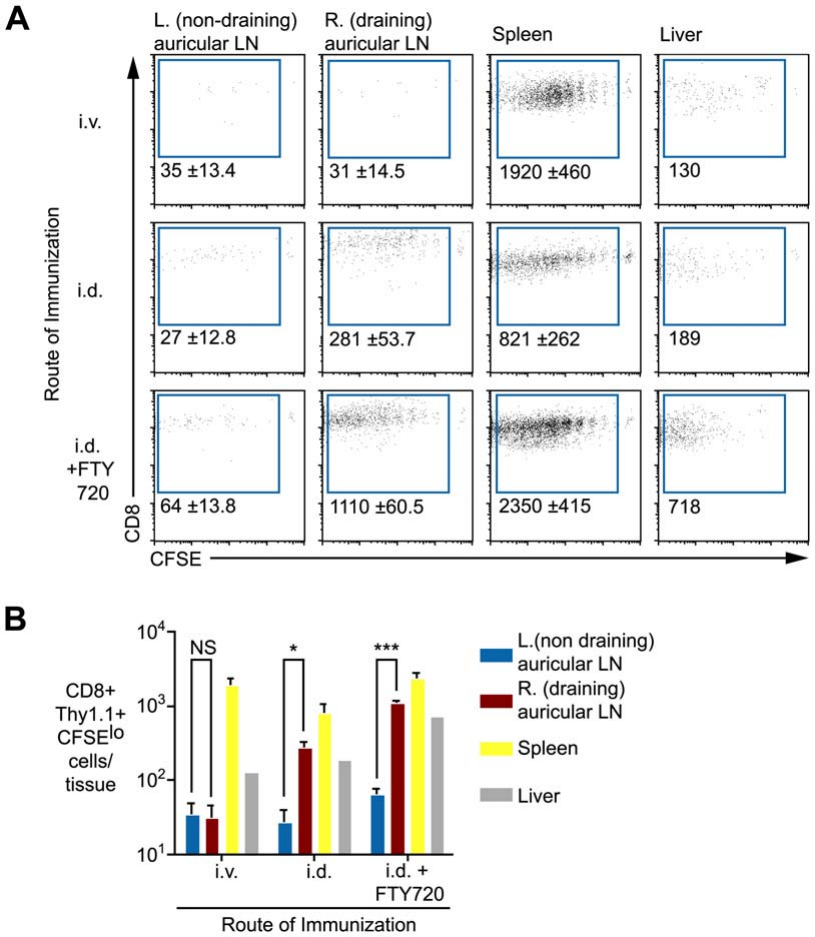
Antigen persists in the draining lymph nodes. A. Mice were immunized i.v. or i.d. with 5×10^4^ irradiated sporozoites. Fourteen days later 1×10^4^ CFSE labeled transgenic cells were transferred to the mice, some of the mice that had been immunized i.d. received daily doses of 30 µg FTY720 after cell transfer to prevent egress of primed T cells. Eight days after cell transfer transgenic cells were enriched from various tissues by positive selection. Flow cytometry plots show representative data from each group. Values are the mean ± standard deviation of the number of divided transgenic cells recovered per tissue of three mice per group. B. Histograms of the data presented in A. (n = 3, mean ± SE, P>0.05, * = P<0.05, *** = P<0.001). Data in A and B are representative of four similar experiments.

Given that antigen does not appear to persist as exo-erythrocytic forms in the liver, we hypothesized that it may be retained in lymphoid organs, particularly those draining the site of immunization. To test this, mice were immunized i.v. or intra-dermally (i.d.) in the right ear. Fourteen days later labeled transgenic cells were transferred to the mice. When sporozoites were injected i.v. significant numbers of dividing T cells were observed in the spleen but not in the lymph nodes of mice (Figure 4A). In contrast, following i.d. immunization, dividing cells were seen in the draining auricular lymph nodes as well as the spleen. Significantly, 10-fold more dividing cells were seen in the draining right auricular lymph nodes compared to the non-draining left auricular lymph nodes (Figure 4A and B), indicating that antigen is retained in the lymphoid tissues where sporozoite antigen is first trapped and presented to naïve cells. In addition we also saw cells dividing in the spleen and liver, the divided cells found in these organs following i.d. immunization could have migrated from the ear draining lymph node or they could have been primed *in-situ*. To distinguish these possibilities another group of mice that received parasites i.d. was given daily injections of 30 µg FTY720 after T cell transfer to prevent the egress of activated T cells from the lymph nodes in which they are primed [22]. In drug treated animals we saw an increased accumulation of activated cells in the draining lymph node, the spleen and the liver (Figure 4A and B) suggesting that the spleen and liver might also be sites of antigen retention. Previously we determined that lymphoid organs, chiefly the skin-draining lymph nodes, but also the spleen, are the principal sites of T cell priming [23] – here we show that antigen is mainly retained in these sites. We also find some evidence of antigen persistence in the liver, which cannot be excluded as a site of T cell priming.

### Professional APCs are required for prolonged antigen presentation

**Figure 5:**
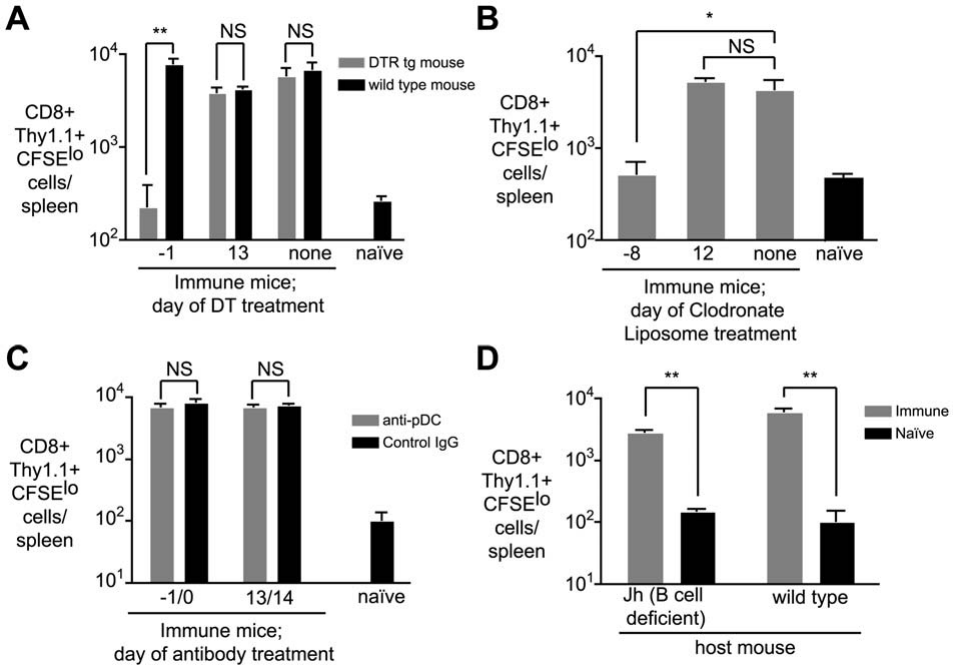
CD11c+ and phagocytic cells are required for continued antigen presentation. A. CD11c-DTR and WT mice were immunized i.v. with 5×10^4^ irradiated sporozoites at day 0. Groups of CD11c-DTR and wild-type mice were treated with 100ng of DT on the indicated days. On day 14, 2×10^5^ CFSE labeled transgenic cells were transferred to the mice. Ten days later, the number of divided transgenic cells was determined by FACs (n = 3, mean ± SE, * = P<0.05, ** = P<0.01, data representative of four experiments). B. Groups of mice were immunized on day 0 as in A and treated with clodronate liposomes (200 µl i.v./mouse) at the indicated days to deplete splenic macrophage populations. On day 14, 2×10^5^ CFSE labeled transgenic cells were transferred to the mice. Ten days later, the number of divided transgenic cells was determined by FACs (n = 3, mean ± SE, * = P<0.05 data representative of two experiments). C. Groups of mice were immunized on day 0 with irradiated sporozoites and treated with 200 µg of the anti-plasmacytoid DC mAb 120G8 or control IgG at the indicated days. On day 14, 2×10^5^ transgenic cells were transferred to the mice. Ten days later the number of divided transgenic cells was determined by FACs. (n = 3, mean ± SE, data representative of two experiments). D. Jh (B cell deficient) and WT mice were immunized with irradiated sporozoites. 14 days later 2×10^5^ transgenic cells were transferred to the mice. Ten days after cell transfer the number of divided transgenic cells in immune mice was determined by FACs and compared to background proliferation of cells transferred to naïve mice (n = 3, mean ± SE, ** = P<0.01, data representative of two experiments).

Since antigen persists in both lymphoid and non-lymphoid tissues we investigated the roles that professional APCs such as myeloid DCs, macrophages or plasmacytoid DCs may play a role maintaining and presenting antigen over the long term. Mice were depleted of APC subsets at one of two time-points – either before immunization on d0 or after immunization and prior to the transfer of transgenic cells on d14 (ie. at the time we detect persistent antigen presentation). To assess the role of DCs we used mice which express the diphtheria toxin receptor under the control of a CD11c promoter; DCs can be depleted in these animals with low doses of diphtheria toxin (DT) [24]. Macrophages were depleted from WT mice using clodronate liposomes (CL) [25] while plasmacytoid DCs were depleted using the mAb 120G8 [26]. DT treatment of CD11c-DTR transgenic mice resulted in ∼90% depletion of CD11c+ cells, while clodronate lipsome depletion induced ∼90% depletion of CD11b^int^ F4/80-, CD11b^int^F4/80+ and CD11b^hi^F4/80+ macrophage populations (Figure S3). Interestingly we found that depletion of CD11c+ cells in DTR mice and treatment with CL had similar effects. In both cases, depletion at the time of immunization eliminated persisting antigen (Figure 5A and B). It may be that DCs and macrophages may be acting co-operatively to trap and retain antigen. Alternatively since the treatments are not mutually exclusive (Figure S3) a single cell type that is depleted by both treatments, for example immature DCs, may be important for antigen persistence.

Most strikingly, however, we found that neither depletion protocol had any effect on prolonged antigen presentation if the treatments were given after immunization (Figure 5 A and B). This may indicate that antigen is transferred to another cell type altogether for presentation. Transfer of antigen between APCs is not unprecedented – herpes simplex virus antigen is trapped by dermal DCs in the skin and transferred to lymph node DCs for presentation [27]. Alternatively the antigen retaining macrophage or DC may continue to present the antigen but become resistant to depletion – a mature APC may become non-phagocytic or downregulate CD11c.

In contrast to CL or DT treatment, administration of the 120G8 antibody had no effect on antigen persistence at any time-point, likely ruling out a role for plasmacytoid DCs in the retention or presentation of persisting antigen (Figure 5C), though the efficacy of depletion was only around 70% (Figure S3). Finally we excluded a role for B cells in antigen persistence since antigen persisted in Jh mice which constitutively lack these cells (Figure 5D [28]).

### Prolonged antigen presentation maximizes the size of the effector CD8+ T cell population and induces proliferation of memory CD8+ T cells

**Figure 6:**
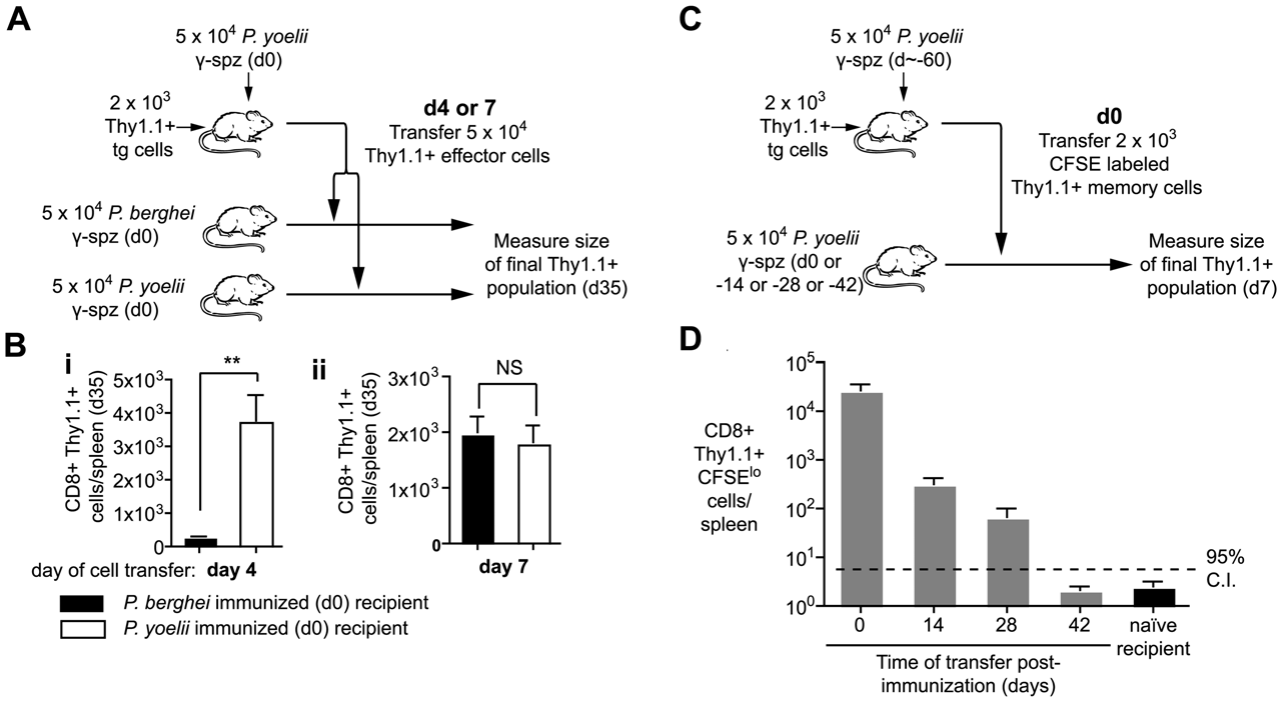
Prolonged antigen presentation is required for optimal CD8+ memory formation. A and B. Naïve mice received 2×10^3^ transgenic cells and were immunized on d0 with 5×10^4^ irradiated *P. yoelii* sporozoites. On either d4 or d7 CD8+ T cells were purified from these mice and the equivalent of 5×10^4^ transgenic cells were transferred to recipients that had received 5×10^4^ irradiated *P. yoelii* sporozoites or 5×10^4^ irradiated *P. berghei* sporozoites on d0. On d35 the size of the transgenic memory population was measured by FACs. A. Outline of the experiment. B. Size of the resulting memory populations after cells transfer on (i) d4, (ii) d7 (n = 5, mean ± SE, ** = P<0.01). C and D. BALB/c mice were immunized with 5×10^4^ irradiated sporozoites and 2×10^3^ d60 memory transgenic cells were transferred to mice at different time points after immunization. Ten days after transfer the transgenic cells were recovered and the number of divided cells was determined C. Outline of the experiment. D Number of divided cells recovered (n = 3, mean ± SE; a positive response is considered > upper 95% confidence interval of the response seen in naïve mice, data from one of two similar experiments).

Prolonged antigen presentation may contribute to the development and maintenance of immune responses in a variety of ways. Initially we asked how long cells had to be exposed to antigen in order to maximize their expansion and development. To do this we transferred 2×10^3^ transgenic cells to mice which were then immunized with irradiated *P. yoelii* sporozoites. Four and seven days after immunization, the primed effector cells were purified and transferred either into mice that had been immunized on d0 with *P. yoelii* and thus continue to see the relevant antigen, or into mice that had been immunized with *P. berghei,* where they are no longer exposed to cognate antigen (Figure 6A; Figure S1). Importantly, by four days after immunization essentially all the Thy1.1 transgenic cells are effector cells that have been primed by antigen and proliferated (Figure S4). A month after cell transfer we measured the magnitude of the resulting memory cell populations in the two groups of mice. When cells were transferred to *P. yoelii* immunized mice at day 4 they formed a memory population that was ∼13 times larger than that formed by cells transferred to *P.berghei* immunized mice (Figure 6B). However, after day 7 the magnitude of the response did not depend on the presence of antigen. The requirement for antigen exposure for 4–7 days was striking as previously it has been reported that T cells require only a brief (<24 hour) exposure for their development [6], [7]. Finally we found that cells that were only exposed to antigen for 4 days had similar cytokine profiles upon re-stimulation to cells continuously exposed to antigen (Figure S5), suggesting that longer antigen presentation is not required for the differentiation of cells but to maximize their expansion.

To determine the effect that long term antigen presentation has on fully differentiated memory cells we transferred d60 memory cells to mice that had received antigen 0–42 days previously (Figure 6C). Differently to effector cells, we found that antigen could induce the proliferation and expansion of d60 memory cells up to 28 days after immunization (Figure 6D). Similar results were obtained with d200 memory cells. In contrast memory cells transferred to naïve mice did not undergo significant proliferation. Thus it appears that the responsiveness of cells to persisting antigen correlates with their activation status – naïve cells are exquisitely sensitive to antigen (Figure 2), while resting memory cells can also turn over in response to antigen. On the other hand beyond 4–7 days of activation effector cells appear to be independent of antigen.

### Priming of thymic emigrants by persisting antigen

**Figure 7:**
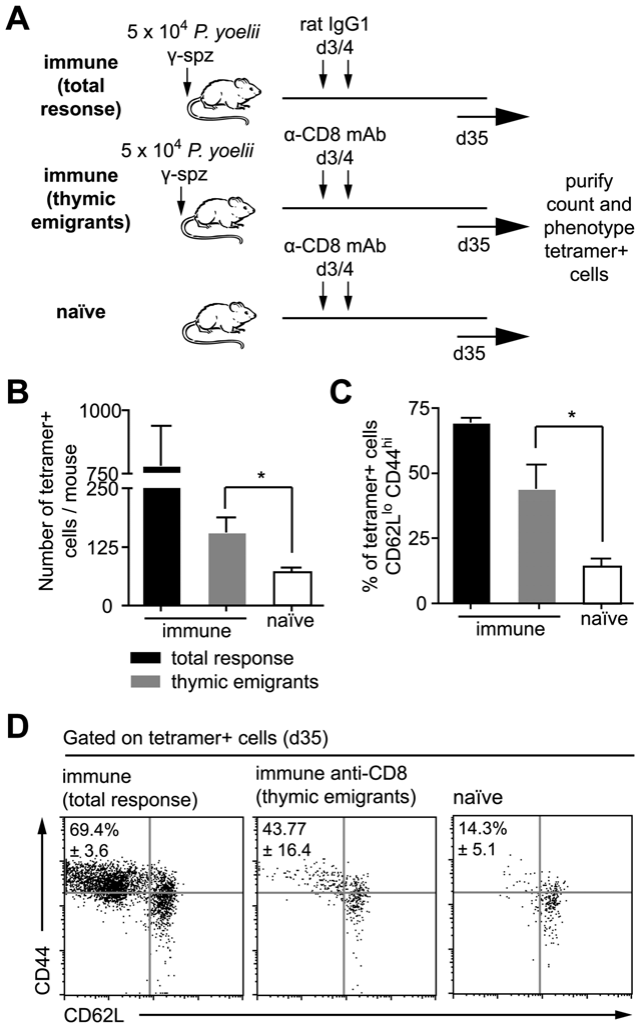
Persisting antigen primes naïve thymic emigrants. A. Outline of the experiment: mice were immunized with 5×10^4^ irradiated sporozoites i.v. On days 3 and 4 after immunization some of the mice were depleted of CD8+ cells such that the observed response consists largely of later thymic emigrants (gray bars). Control mice were treated with control IgG1 leaving the pre-existing response in tact (total response – black bars), while other mice were left un-immunized but treated with anti-CD8 (naïve – white bars). B. Histograms of the number of tetramer+ cells recovered in each group, bar charts show means ± SE (n = 6, * = P<0.05, data pooled from two similar experiments). C. Histograms of the percentage of activated cells (CD62L^lo^, CD44^hi^) among the tetramer+ population recovered in each experiment (n = 3, * = P<0.05, data from one of two similar experiments). D. Dot plots of the CD62L and CD44 expression of tetramer+ cells in each group, data show concatenated data from 3 animals per group and are from one of two similar experiments.

The previous experiments demonstrate that a prolonged period of antigen presentation throughout the expansion phase of the CD8+ T cell response is required to maximize the CD8+ T cell response. However antigen continues to be presented for weeks after this. In addition to showing that this late antigen presentation could stimulate the proliferation of memory cells we also hypothesized that prolonged antigen presentation may continue to recruit naïve cells, such as naïve thymic emigrants to the immune response. To test this we immunized mice with sporozoites and 3 days later depleted CD8+ T cells by antibody treatment. Preliminary experiments showed that this treatment has no effect on the presence of persisting antigen (data not shown). Around 10 days after depletion, CD8+ T cells begin to reappear, these repopulating cells are, in all likeliness, mostly thymic emigrants (Figure 7A). Thirty-five days after immunization we isolated the endogenous tetramer+ cells by magnetic bead selection and stained for the surface markers CD44 and CD62L. We found antigen specific thymic emigrants from immune mice expanded ∼2.5-fold compared to cells in naïve mice - a difference that was significant (Figure 7B; P = 0.027 by two-tailed T test; t = 2.58; df = 10). More importantly, thymic emigrants had up-regulated CD44 and down-regulated CD62L, a phenotype similar to that seen in immunized mice which had received control IgG instead of anti-CD8 (Figure 7C and 7D) suggesting that thymic emigrants are indeed primed by persisting antigen.

It is possible that some of the tetramer+ activated cells we see after depletion are not thymic emigrants but cells that survived anti-CD8 depletion and subsequently expanded by homeostatic proliferation. To estimate the likely percentage of such cells we transferred 2×10^3^ transgenic Thy1.1+ cells to two further groups of Thy1.2+ mice that were immunized with sporozoites. We then compared the ratio of Thy1.1+ transgenic and Thy1.2+ endogenous tetramer+ cells in CD8+ depleted and non-depleted animals. If the activated cells we see after depletion are survivors of this depletion we would not expect this ratio to change, on the other hand if few cells survive the depletion then a smaller proportion of the cells will be Thy1.1+ as these cells can not be replaced by thymic emigrants. The ratio of transgenic cells:endogenous cells was 1.2∶1 in depleted mice but 6.9∶1 in undepleted animals (Figure S6), a significant difference from what would be expected if the tetramer+ cells in CD8+ treated animals were only depletion survivors (P<0.0001 by Fisher's exact test). By comparing these ratios (1.2/6.9) we estimate that ∼17% (95% CI: 12–27%) are likely to be depletion survivors whereas >80% of the response can likely be attributed to thymic emigrants.

### Persisting antigen can induce naïve T cells to differentiate into functional effector and memory T cells

**Figure 8:**
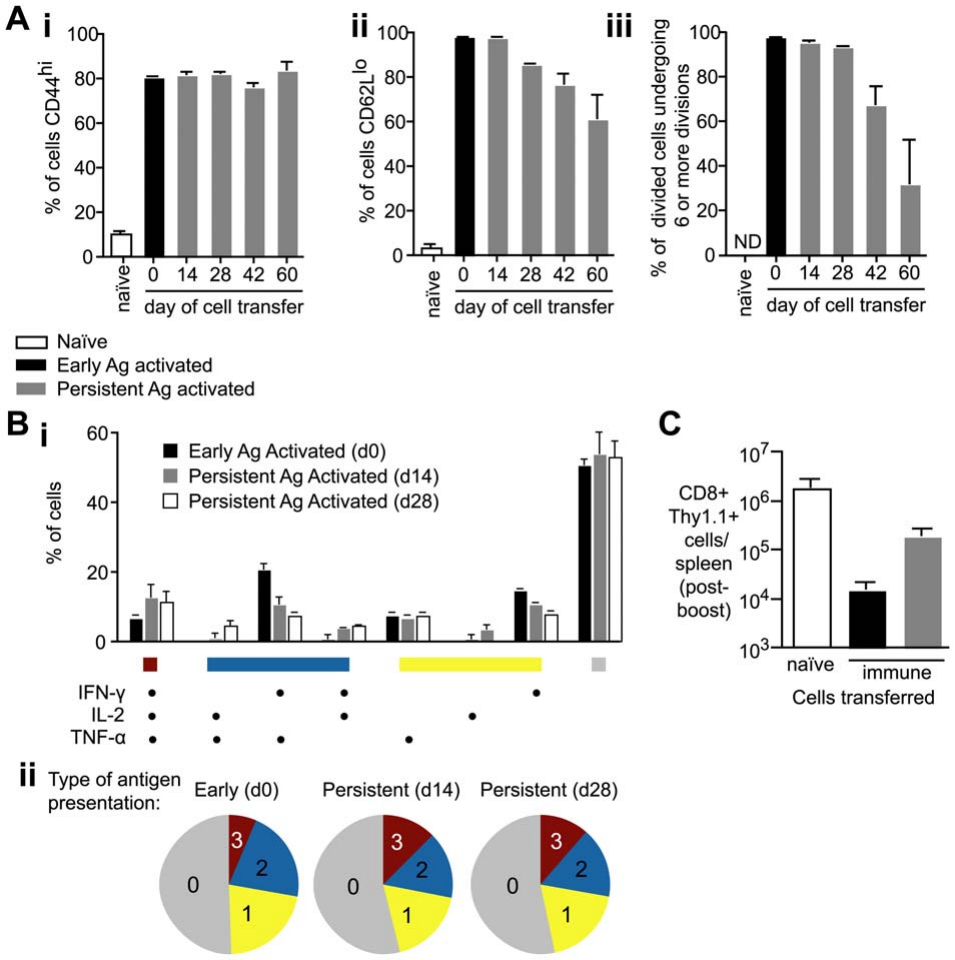
Antigen presentation weeks after immunization can prime T cells to become functional effector cells. A. 2×10^3^ CFSE labeled transgenic cells were transferred to mice that had been immunized 14, 28, 42 and 60d previously with 5×10^4^ irradiated sporozoites i.v. (persistent-antigen activated cells – gray bars). Phenotypes of persistent antigen activated cells were compared to the phenotypes of early antigen activated cells, in which 2×10^3^ transgenic cells were transferred to naïve mice and immunized the same day with 5×10^4^ irradiated sporozoites (black bars). Naïve control mice received 2×10^6^ CFSE labeled transgenic cells. 7 days after cell transfer transgenic cells were isolated and analyzed for (i) CD44 expression, (ii) CD62L expression, and (iii) CFSE dilution (n = 3, means ± SE, ns = not significant, * = P<0.05, ** P<0.01, data from four similar experiments). B. Polyfunctional analysis comparing the cytokine expression of cells stimulated by early (d0) or persisting antigen (d14 or d28) 35 days after cell transfer. i. histogram shows the % of transgenic cells producing each possible combination of cytokines in response to early antigen presentation (black bars), d14 antigen presentation (gray bars) or d28 antigen presentation (white bars) ii. pie charts representing the number of cells producing 3 (red), 2 (blue), 1 (yellow) or 0 (gray) cytokines (data from three similar experiments). C. 2×10^3^ naïve transgenic cells (white bar), 2×10^3^ d35 memory transgenic cells primed by early antigen (black bar) and 2×10^3^ d35 memory transgenic cells primed by persisting antigen (gray bar) were transferred to naïve BALB/C mice which were immunized with 2×10^6^ VV-SYV i.v., 14 days later the number of Thy1.1+ transgenic cells was measured by FACs (n = 3, mean ± SE, data from two similar experiments).

We further wished to determine whether primed thymic emigrants could become functional effector and memory cells. However we were limited by the small number of cells in the endogenous response. To mimic the response of thymic emigrants we transferred 2×10^3^ naïve transgenic cells to mice that were immunized 14–60 days previously and compared the phenotypes of these cells (persistent antigen primed cells) to those primed by immunization on the same day as cell transfer (early antigen primed cells). Seven days after cell transfer we measured the surface phenotypes of transgenic cells. T cell populations primed at all time points had significantly upregulated CD44 and downregulated CD62L suggesting that persisting antigen can drive the cells to form an effector population (Figure 8A i and ii), however some cells primed at day 42 and 60 had not down regulated CD62L as much as cells primed at earlier time points (Figure 8A ii). These cells may be less differentiated than cells primed earlier in the response as indicated by the fact that fewer cells had undergone 6 or more divisions (Figure 8A iii). Crucially, however, when we compared the polyfunctional cytokine profile 35 days after cell transfer we found no significant differences in the functionality of the cells: production of IFN-γ, TNF-α or IL-2 was similar regardless of whether those cells had been primed early in the response or by persisting antigen on d14 or d28 (Figure 8B). To verify that the cells primed by persisting antigen could be bona-fide memory cells we tested their ability to mount recall responses. When 2×10^3^ persistent antigen primed cells were transferred to naïve mice and boosted with a vaccinia virus expressing the SYVPSAEQI epitope they expanded better than memory cells primed by early antigen (Figure 8C).

### Cells primed weeks after immunization can inhibit parasite development

**Figure 9:**
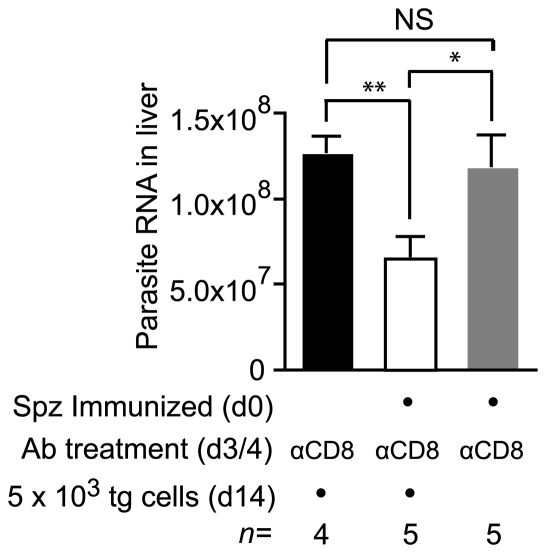
Cells primed weeks after immunization can inhibit parasite development. Two groups of mice were immunized with 3×10^4^ irradiated sporozoites i.d. in the right ear on d0 and depleted of the endogenous CD8+ T cell response on days 3 and 4. Some of these mice received 2×10^3^ transgenic cells on d14 (white bar) while others did not receive cells (gray bar). Naïve control mice were similarly depleted of CD8+ T cells on days 3 and 4 and received transgenic cells (black bar). All mice were then challenged on d21 and euthanized 40 hours later. Parasite burdens in the liver were measured by RT-PCR (mean ± SE, * = P<0.05, ** = P<0.01, data from one of two experiments shown).

To determine if persistent antigen primed cells could develop anti-parasite acitivity we transferred transgenic cells to mice that had been immunized 14 days previously. Mice that received transgenic cells had been depleted of CD8+ cells on days 3 and 4 after immunization, to ensure that the only sporozoite specific effector CD8+ cells present in these mice would have been primed by persisting antigen. Seven days after cell transfer the mice were challenged with live *P. yoelii* sporozoites and the parasite load measured in the liver. Compared to naïve control mice - which had also been depleted of CD8+ T cells and received transgenic cells – parasite development in immunized mice was inhibited by approximately 50%. This level of anti-parasite activity was significant (P = 0.0016 by two-tailed T test; t = 4.96; df = 7), demonstrating that CD8+ cells primed by persisting antigen can develop anti-parasite effector mechanisms and are likely to contribute to protective immunity (Figure 9). Importantly, parasite killing could not be attributed to antibodies, CD4+ T cells or CD8+ T cells that survived depletion, since immunized mice that were treated with anti-CD8 but did not receive transgenic cells were infected just as readily as naïve control animals (Figure 9).

## Discussion

In this study we show that CS antigen continues to be presented for nearly 2 months following irradiated sporozoite immunization – a striking result given that irradiated sporozoites are incapable of replication and only transform abortively into early liver stages [29], [30]. Sporozoite antigen presented weeks after immunization could prime naïve T cells to acquire effector functions and become capable of eliminating liver stage parasites. Importantly, curtailing antigen presentation by transferring specific effector T cells to mice without persisting antigen profoundly reduced the size of the resulting memory cell pool.

The presence of persisting antigen following sporozoite immunization has been proposed previously based on the continuing presence of parasite remnants in the liver [5] though the actual presence of antigen was not proven. In showing that antigen specific cells proliferate in immunized but not control mice, and that this effect cannot be attributed to inflammation, we demonstrate for the first time that antigen does indeed persist after sporozoite immunization. The use of a functional assay to detect antigen is not only more definitive than detection by immunohistochemistry, but probably also more sensitive as T cells can proliferate in response to a single peptide-MHC Class I complex [31]. Given our findings that (i) antigen persists in lymphoid tissue, (ii) DCs and/or macrophages are required to establish antigen persistence and (iii) antigen persistence does not appear to be dependent upon the continuing presence of parasite forms in the liver it is probable that the antigen persistence we observe is a distinct phenomenon from that previously described. Indeed in our system antigen may be persisting, not as parasite forms but as protein or even MHC Class I complexes.

Because we describe a distinct aspect of sporozoite biology our work does not directly address the controversial issue of whether long-lived parasite forms in the liver are required to maintain protection. In many studies PQ treatment to remove such exo-erythrocytic remnants has been used to ablate protective immunity after sporozoite immunization, [5], [32], [33], [34]. However, other investigators, including ourselves, have been unable to see an effect of PQ on CD8+ T cell responses and immunity [35], [36], [37] (Figure S7). Moreover, our data do not support the notion that PQ treatment removes persistent antigen and thus abrogates immune responses. In at least some experiments PQ was administered hours after immunization with parasites [32], [34] and its effect may be due to the inhibition of responses to later liver stages or blood stages rather than any effect on remnant parasite forms in the liver. Alternatively since PQ is a rather toxic drug [38] it may be acting ectopically to inhibit immune responses.

These findings have profound implications for our understanding of immunity after vaccination with irradiated sporozoites. The irradiated sporozoite model of immunization against malaria is one of the few vaccine models where CD8+ T cells play a major protective role as T cells specific for a single epitope can completely eliminate parasite infection in the liver [39]. Antigen persistence probably plays a significant role in the development of these protective CD8+ T cells. We found that after immunization with heat killed, irradiated or live sporozoites antigen persistence correlates with the ability to induce protective immune responses: heat killed sporozoites induce very poor CD8+ T cell responses [40], while both irradiated sporozoites and live sporozoites induce robust protective CD8+ T cell responses [2], [34]. While live or attenuated sporozoites may potentiate immunity in a variety of ways, for example by generating more antigen or eliciting danger signals, our findings that prolonged antigen presentation recruits cells to immune response and increases the number of memory cells show that the ability to induce prolonged antigen presentation may be a requirement for effective vaccination.

Our finding that prolonged antigen presentation is required for maximal CD8+ T cell expansion and memory formation apparently contrasts with experiments using different systems, which suggest that only a short burst of antigen presentation is required for full differentiation of CD8+ T cells [6], [7], [8]. How can these differing results be reconciled? One explanation for differences between studies is that powerful replicating immunogens such as *Listeria* expressing ample danger signals such as TLR ligands may be capable of efficiently priming T cells in a short period of time. In contrast, *Plasmodium* sporozoites, a non-dividing immunogen, may have more stringent requirements for T cell priming, including a need for prolonged antigen presentation. Alternatively, we might propose that antigen dose and time of exposure are important for the magnitude of the immune response, but, above a minimal threshold, may not be critical for the quality of T cell activation. The uncoupling of T cell differentiation from T cell expansion would also explain why T cells primed by small amounts of persisting antigen can differentiate similarly to T cells primed by larger amounts of early antigen without the same degree of T cell expansion.

In addition to being important for the maximal proliferation of effector cells, persisting antigen can recruit naïve cells to the immune response for at least 60 days after immunization and induce proliferation of established memory cells. It has been previously shown that thymic emigrants contribute significantly to the immune response to chronic viral infections [14], [15] – here we show this process may also be important in the absence of ongoing infection. Further work will be required to determine the relative significance of the cells primed late compared to those primed soon after immunization. It may be that the T cells primed in the initial 4–7 days of antigen exposure are sufficient to establish immunity. Alternatively cells primed late in the immune response may have a role in the development of memory populations – particularly, perhaps, central memory populations since in our experiments late primed cells were more likely to be CD62L^hi^ and expanded more readily upon boosting than early primed cells.

In addition to the roles prolonged antigen presentation plays in maximizing the T cell response to immunization, our data show that antigen persists following infection with live parasites. This may have important implications for immune responses in endemic areas where people are exposed to sporozoites on a daily to monthly basis; if antigen is retained similarly in humans it would mean many people would be permanently exposed to sporozoite antigen [41]. There is some evidence that antigen persistence has a positive role in these circumstances – T cell responses to *P. vivax* which forms long lived hypnozoite stages in the liver are generally more robust and long lived than responses to *P. falciparum*, at least among travelers and individuals in areas of low transmission [42], [43]. However, paradoxically, given our results showing that antigen persistence has a positive role in the development of immune responses, T cell responses to sporozoite antigens are generally considered rather poor in individuals in endemic areas [44]. A variety of reasons have been given for limited responses in endemic areas: we have shown that once established CD8+ T cells have powerful feedback regulatory mechanisms that limit T cell expansion. Under these conditions the magnitude of a T cell response may be set by initial antigen exposure, which in the case of mosquito bites will be small [35], [45]. Thus while continued antigen presentation may recruit cells to the sporozoite specific immune response, the expansion of these cells may be continually regulated by established effector and memory cells which compete with naïve cells for antigen. Since initial exposure is the most important determinant of the threshold at which specific CD8+ T cell responses are set, it is not surprising that a single immunization with a very high number of irradiated sporozoites would give a stronger response than natural exposure.

Using similar experimental approaches to those described here antigen persistence has been studied following acute influenza and vesicular stomatitis virus infections [16], [17], [18], [19]. The process of antigen retention is probably analogous between these systems and ours: in all cases antigen is retained in lymphoid tissue in the absence of detectable infection for a period of 1–2 months. Nonetheless our results showing efficient formation of T cell memory among cells primed by persisting antigen contrast with some findings in other systems. Some studies using influenza virus infection have suggested that persistent antigen presentation may result in sub-optimal priming of naïve T cells [19], [46]. Such defects might be explained by differences in the processing of sporozoite and viral antigen. However it is also notable that all other studies detecting persisting antigen using transgenic T cells have employed the transfer of high (2×10^5^–1×10^6^) numbers of naïve transgenic cells. In preliminary experiments we also used un-physiological numbers of transgenic cells and found evidence of defective T cell priming, including the fact that <15% of divided cells produced any effector cytokine (Figure S8). If the differences between our findings and those of other authors are based on experimental rather than biological differences it maybe that antigen retention is a common feature of the immune response to many live pathogens.

Determining the cells involved in retaining and presenting persisting antigen is an area for further investigation. Professional antigen presenting cells clearly play a role in prolonged antigen presentation. Both CD11c+ cell depletion in DTR transgenic mice and CL treatment in WT mice at the time of immunization almost completely abrogated antigen persistence. These data strongly suggest a role for CD11c+ DCs and macrophages in antigen persistence. However since DT treatment also removes some macrophages and clodronate liposomes can deplete some DCs (Figure S3; [47]) it is possible that only a single cell type that is depleted by both treatments is important for prolonged antigen presentation. Strikingly, we were unable to deplete the cells that actually present antigen weeks after immunization. The simplest explanation for these data would be that CD11c+ cells acquire antigen and transfer it to another depletion resistant cell for continued presentation. Transfer of antigen between cell populations has been described previously in a variety of systems so this is not implausible [27], [48], [49]. Lymphoid stromal cells which have been shown to be capable of presenting self antigens could be one candidate recipient cell population [50]. Some studies suggest that stromal cells can acquire antigen from B cells and develop into follicular dendritic cells (FDCs) [51], an analogous process could be occurring for T cell antigen. In our system, mature FDCs themselves are unlikely to be the presenting cell type as these cells are not apparently found in the absence of B cells [52], while we detect antigen persistence in B cell deficient animals. Alternatively rather than transferring antigen to another cell type, DCs or macrophages might retain antigen themselves but become refractory to depletion, this may happen if an antigen retaining cells down-regulate expression of CD11c or phagocytic processes. Nonetheless, the idea of long-lived antigen presenting DCs contradicts the prevailing view that DCs generally undergo rapid turnover [53]. Given that cells retaining antigen are rare and hard to isolate, identifying the relevant cell type is difficult using existing techniques and assays.

Together these data show that antigen may persist following immunization with a non-replicating immunogen. This antigen persistence appears able to enhance the immune response to pathogens by continually recruiting naïve cells into the protective immune response and maximizing the expansion of pathogen specific effectors. These observations clearly have important implications for the design of vaccines particularly those based on attenuated or non-replicating pathogens.

## Materials and Methods

### Ethics statement

All animal procedures were approved by the Institutional Animal Care and Use Committee of the Johns Hopkins University (Protocol Number MO09H41) following the National Institutes of Health guidelines for animal housing and care.

### Mice

5–8 week old female BALB/C were purchased from Taconic (Hudson, NY). Transgenic mice expressing a TCR specific for the *P. yoelii* epitope were derived as previously described [54]. Mice from our colony that have previously been backcrossed to the Thy1.1^+^ BALB/C background for >20 generations were used. CD11c-DTR mice on a BALB/C background were derived as described and are maintained in our colony as heterozygotes [24]. Jh mice deficient in have been described previously [28] and were purchased from Taconic. Experiments involving mice were approved by the institutional animal care and use committee of the Johns Hopkins University.

### Immunizations and drug treatment

*P. yoelii* 17X NL and *P. berghei* ANKA sporozoites were obtained and irradiated as previously described [54]. The dose of radiation required to fully attenuate sporozoites was 40kRad for *P. yoelii* and 20kRad for *P. berghei*. Immunizations (5×10^4^ sporozoites unless otherwise stated) were given i.d. in the right ear where possible [23]. In some experiments it was necessary to use i.v. immunization to obtain enough material from the spleen for phenotypic analysis; i.v. immunization was also necessary in depletion experiments as treatments such as clodronate liposomes do not deplete efficiently in peripheral tissues when given systemically [25]. Sporozoites were heat killed by incubation at 65°C for 15 minutes. To prevent blood stage infections following live sporozoite infection mice were treated from 40 hours to 7 days after immunization with pyrimethamine at 70 µg/ml in the drinking water. In some experiments PQ was administered in 2 doses of 60mg/kg s.c., 10 hours apart 7 days after sporozoite immunization; this treatment is effective but highly toxic, of 10 control mice infected with 5×10^3^ *P. yoelii* sporozoites and treated with 60mg/kg PQ 10 and 20 hours after challenge, 4 died of drug treatment though the remaining 6 did not develop parasitemia.

### Quantification of parasite RNA

Quantification of liver stage parasites was as previously described [55]. Briefly, 40–42 hours after challenge, livers were excised and parasite load was determined by quantitative PCR for *P. yoelii* 18S rRNA using SYBR Green (Applied Biosystems).

### Cell depletion

DCs were depleted from CD11c-DTR transgenic mice by 2 i.p. injections of 100ng DT 3 days apart. This resulted in approximately 90% depletion of DCs for around 120 hrs. BALB/C mice are able to accept this dose without weight loss or the mortality seen in C57/B6 mice [56]. CD8+ cells were depleted by 2 i.p. injections on consecutive days with 200 µg anti-CD8 (clone 2.43). Clodronate liposomes were prepared and used as described [25], clodronate was a gift of Roche Diagnostics GmbH, Mannheim, Germany. To deplete plasmacytoid DCs mice were treated on consecutive days with 100 µg of the rat mAb 120G8 as described previously [26].

### Cell isolation and preparation of samples for flow cytometry

Single cell suspensions of lymphocytes were obtained by grinding spleen cells or lymph node cells between the ground ends of two microscope slides and filtering twice through 100 µm nylon mesh. Liver lymphocytes were isolated from perfused livers by grinding, filtration through a 70 µm mesh and separation over a 35% percoll gradient as described [57]. Where necessary, untouched purified populations of CD8+ T cells were obtained using the mouse CD8+ T cell isolation kit according to the manufacturers instructions (Miltenyi Biotech, Auburn, CA). Rare antigen specific cell populations were enriched as previously described [58], [59], [60]. Briefly cells were incubated with anti-Thy1.1-PE (if transgenic cells were being enriched) or Tetramer-PE (if the endogenous population was being examined). The cells were then positively selected by magnetic bead separation using anti-PE microbeads and LS columns (Miltenyi Biotech). To identify the antigen specific cells of interest the cells were co-stained with CD8, while irrelevant cells were excluded by staining with a dump gate pool of CD11b, CD4 and B220. To analyze the expression of T cell activation markers cells were also stained simultaneously with anti-CD44, CD27 and CD62L and analyzed on a LSR-II flow cytometer (BD Biosciences).

### Polyfunctional cytokine assay

For analysis of T cell functionality based on cytokine production, cells were incubated with peptide-coated target cells in the presence of protein transport inhibitors and then stained for intracellular cytokines. Briefly, A20 target cells were pulsed with SYVPSAEQI peptide (2 µg/mL) and control A20 cells were incubated without peptide. Peptide-coated or control target cells (6×10^5^ per well) were added to a 96-well V-bottom plate with 2×10^6^ effector cells. To measure cytokine production, cells were incubated with brefeldin A (GolgiPlug, BD Biosciences) to block protein transport. Cells were stimulated for 5 hours at 37°C and then washed twice in cold media and then surface stained with anti-CD8 (clone 53–6.7) and anti-Thy-1.1 or dump gate antibodies as required. Cells were then permeabilized and fixed using a Cytofix/Cytoperm kit (BD Biosciences) according to the manufacturer's instructions and stained for intracellular cytokines using anti-IFN-γ (clone XMG1.2), anti-TNF-α (clone MP6-XT22), or anti-IL-2 (clone JES6-5H4, all from eBiosciences) at pre-determined concentrations. Cells were then washed and analyzed on a LSR II flow cytometer (BD Bioscience).

### Data analysis

Statistical analysis was performed using Prism 4 software (GraphPad Software), unless otherwise stated, means were compared by two-tailed Student's t tests. Analysis of all FACS data was performed using FlowJo software (TreeStar). Seven color samples were compensated using matrices generated with the single color controls and FlowJo software. For analysis of polyfunctionality, single gates were drawn for IFN-γ+, TNF-α+, and IL-2+ cells among CD8+Thy-1.1+ lymphocytes (gates were set using control non-stimulated transgenic cells). Boolean combination gates were then created to generate the 8 possible functional groups from the 3 individual functions. Data was exported to PESTLE and SPICE (both kindly provided by Mario Roederer) for analysis.

## Supporting Information

Figure S1 Proliferation of transgenic cells is not due to an ongoing inflammatory response. A. 2 × 10^6^ Thy1.1+ CD8+ T cells from TCR transgenic were transferred into mice immunized 2 weeks previously with either 5 × 10^4^ irradiated *P. yoelii* sporozoites or 5 × 10^4^ irradiated *P. berghei* sporozoites. 10 days after cell transfer the spleen cells from the recipient mice were analyzed by FACs. Histograms show representative CFSE profiles of the Thy1.1+ CD8+ cell populations in each group. (n  =  3, data from one of two similar experiments shown). B. As in A except only 2 × 10^3^ transgenic cells were transferred.
(2.25 MB TIF)

Figure S2 Identification of rare transgenic cell populations. 2 × 10^3^ CFSE labeled transgenic cells were transferred to mice that had been immunized 14 days previously with 5 × 10^4^ irradiated sporozoites. 10 days later the mice were sacrificed and the spleens taken. The cells were stained with anti-Thy1.1 PE and then incubated with anti PE-microbeads (Milteyi Biotech) before positive selection using an LS column. Cells were then counter stained with anti CD11b, anti-CD4 and anti-B220 (all conjugated to PE-Cy7) and anti-CD8 APC and analyzed by FACs. Rare transgenic cells could then be identified using the gating protocol shown and the extent of proliferation determined from CFSE dilution.
(1.71 MB TIF)

Figure S3 Depletion of dendritic cell and macrophage subsets. A. CD11c-DTR mice were treated with 100ng DT or PBS as a control. One day later the efficacy of depletion was assessed by FACs. Plots show (i) % of cells CD11c+ DTR-GFP+ (gated on CD3- granulocytes) and (ii) partial depletion of macrophage subsets in DT treated animals, (gated on CD11c- CD3- granulocytes). B. BALB/C mice were treated once with 200μl clodronate liposomes, 8 days later the depletion of macrophage subsets was assessed by FACs. Plots show (i) the depletion of CD11b+ and F4/80+ subsets (gated CD11c- CD3- granulocytes) and (ii) partial depletion of CD11c+ cells (gated on CD3- granulocytes). C. BALB/C mice were treated on consecutive days with 200μg120G8 or Rat IgG. One day later the efficacy of depletion of CD11c+ B220+ plasmacytoid DCs was assessed by FACs (plots gated on CD11c+ granulocytes).
(5.74 MB TIF)

Figure S4 Proliferation of cells four days after immunization. 2 × 10^3^ CFSE labeled Thy1.1 transgenic cells were transferred to mice which were immunized with 5 × 10^4^ *P. yoelii* irradiated sporozoites. Four days later the Thy1.1+ cells were enriched as described and the proliferation of T cells was assessed by CFSE dilution and compared to naïve controls (representative data from one of three mice per group shown).
(1.09 MB TIF)

Figure S5 Polyfunctional profiles of cells transferred to *P. yoelii* and *P. berghei* immunized mice. Polyfunctional analysis of transgenic T cells in Figure 6Bi: Naïve mice received 2 × 10^3^ transgenic cells and were immunized on d0 with 5 × 10^4^ irradiated *P. yoelii* sporozoites. On either d4 CD8+ T cells were purified from these mice and 5 × 10^4^ transgenic cells were transferred to recipients that had received 5 × 10^4^ irradiated *P. yoelii* sporozoites or 5 × 10^4^ irradiated *P. berghei* sporozoites on d0. On d35 polyfunctional analysis was performed on transgenic T cells recovered from these mice. A. Histograms showing the polyfunctional profile of transgenic cells that had been transferred to *P. berghei* immunized (black bars) and *P. yoelii* immunized mice (white bars) 4 days after priming (n = 3, mean ± SE). B. Pie charts showing the number of transgenic cells producing 3, (red), 2 (blue), 1 (yellow) or 0 (gray) cytokines.
(3.03 MB TIF)

Figure S6 Activated cells seen after CD8+ T cell depletion likely represent recent thymic emigrants. Mice received 2 × 10^4^ Thy1.1+ transgenic cells prior to immunization with 5 × 10^4^ irradiated sporozoites. On days 3 and 4 after immunization the mice were treated with either anti-CD8+ mAbs or rat IgG1 as a control. 35 days after immunization the magnitude of the tetramer +ve response was determined and the proportion of Thy1.1+ cells was determined in both groups. Data show the mean ± SE of the number of tetramer +ve cells per group based on 3 mice per group; this experiment was performed concurrently with that shown in Figure 7.
(2.35 MB TIF)

Figure S7 Primaquine treatment does not affect parasite killing in sporozoite immunized mice. Mice were immunized i.v. with 5 × 10^4^ irradiated sporozoites (gray bars) and challenged 37 days later with 5 × 10^3^ live sporozoites with or without primaquine treatment (as in A) on day 7. 40 hours after challenge parasite load was measured in the liver by RT-PCR and compared to naïve controls (black bars; n  =  3, mean ± SE).
(1.20 MB TIF)

Figure S8 Transgenic cells do not acquire full effector function in response to persisting antigen when high numbers of precursors are used. 2 × 10^6^ CFSE labeled transgenic cells were transferred to mice that had been immunized 2 weeks previously with 5 × 10^4^ irradiated sporozoites or to naïve mice which were immunized the same day as transfer. 10 days later the spleens were taken, re-stimulated with peptide and polyfunctional analysis performed. A. The percent of CFSE^lo^ cells producing each effector cytokine (representative data of one of three mice per group, gated on Thy1.1+ CD8+ T cells). B. i histograms showing the polyfunctional profile of transgenic cells that had encountered early (black bars) and persistent (gray bars) antigen presentation (n = 3, mean ± SE) and ii pie charts showing the number of CFSElo transgenic cells producing 3, (red), 2 (blue), 1 (yellow) or 0 (gray) cytokines. Representative data from two similar experiments shown.
(5.44 MB TIF)

## References

[1] Clyde DF (1975). Immunization of man against falciparum and vivax malaria by use of attenuated sporozoites.. Am J Trop Med Hyg.

[2] Nussenzweig RS, Vanderberg J, Most H, Orton C (1967). Protective immunity produced by the injection of x-irradiated sporozoites of plasmodium berghei.. Nature.

[3] Schofield L, Villaquiran J, Ferreira A, Schellekens H, Nussenzweig R (1987). Gamma interferon, CD8+ T cells and antibodies required for immunity to malaria sporozoites.. Nature.

[4] Weiss WR, Sedegah M, Beaudoin RL, Miller LH, Good MF (1988). CD8+ T cells (cytotoxic/suppressors) are required for protection in mice immunized with malaria sporozoites.. Proc Natl Acad Sci U S A.

[5] Scheller LF, Azad AF (1995). Maintenance of protective immunity against malaria by persistent hepatic parasites derived from irradiated sporozoites.. Proc Natl Acad Sci U S A.

[6] Kaech SM, Ahmed R (2001). Memory CD8+ T cell differentiation: initial antigen encounter triggers a developmental program in naive cells.. Nat Immunol.

[7] van Stipdonk MJ, Lemmens EE, Schoenberger SP (2001). Naive CTLs require a single brief period of antigenic stimulation for clonal expansion and differentiation.. Nat Immunol.

[8] Mercado R, Vijh S, Allen SE, Kerksiek K, Pilip IM (2000). Early programming of T cell populations responding to bacterial infection.. J Immunol.

[9] Bevan MJ, Fink PJ (2001). The CD8 response on autopilot.. Nat Immunol.

[10] Day CL, Kaufmann DE, Kiepiela P, Brown JA, Moodley ES (2006). PD-1 expression on HIV-specific T cells is associated with T-cell exhaustion and disease progression.. Nature.

[11] Zajac AJ, Blattman JN, Murali-Krishna K, Sourdive DJ, Suresh M (1998). Viral immune evasion due to persistence of activated T cells without effector function.. J Exp Med.

[12] Callan MF (2003). The evolution of antigen-specific CD8+ T cell responses after natural primary infection of humans with Epstein-Barr virus.. Viral Immunol.

[13] Gillespie GM, Wills MR, Appay V, O'Callaghan C, Murphy M (2000). Functional heterogeneity and high frequencies of cytomegalovirus-specific CD8(+) T lymphocytes in healthy seropositive donors.. J Virol.

[14] Snyder CM, Cho KS, Bonnett EL, van Dommelen S, Shellam GR (2008). Memory inflation during chronic viral infection is maintained by continuous production of short-lived, functional T cells.. Immunity.

[15] Vezys V, Masopust D, Kemball CC, Barber DL, O'Mara LA (2006). Continuous recruitment of naive T cells contributes to heterogeneity of antiviral CD8 T cells during persistent infection.. J Exp Med.

[16] Jelley-Gibbs DM, Brown DM, Dibble JP, Haynes L, Eaton SM (2005). Unexpected prolonged presentation of influenza antigens promotes CD4 T cell memory generation.. J Exp Med.

[17] Turner DL, Cauley LS, Khanna KM, Lefrancois L (2007). Persistent antigen presentation after acute vesicular stomatitis virus infection.. J Virol.

[18] Zammit DJ, Turner DL, Klonowski KD, Lefrancois L, Cauley LS (2006). Residual antigen presentation after influenza virus infection affects CD8 T cell activation and migration.. Immunity.

[19] Jelley-Gibbs DM, Dibble JP, Brown DM, Strutt TM, McKinstry KK (2007). Persistent depots of influenza antigen fail to induce a cytotoxic CD8 T cell response.. J Immunol.

[20] Khan ZM, Vanderberg JP (1991). Role of host cellular response in differential susceptibility of nonimmunized BALB/c mice to Plasmodium berghei and Plasmodium yoelii sporozoites.. Infect Immun.

[21] Badovinac VP, Haring JS, Harty JT (2007). Initial T cell receptor transgenic cell precursor frequency dictates critical aspects of the CD8(+) T cell response to infection.. Immunity.

[22] Pinschewer DD, Ochsenbein AF, Odermatt B, Brinkmann V, Hengartner H (2000). FTY720 immunosuppression impairs effector T cell peripheral homing without affecting induction, expansion, and memory.. J Immunol.

[23] Chakravarty S, Cockburn IA, Kuk S, Overstreet MG, Sacci JB (2007). CD8+ T lymphocytes protective against malaria liver stages are primed in skin-draining lymph nodes.. Nat Med.

[24] Jung S, Unutmaz D, Wong P, Sano G, De los Santos K (2002). In vivo depletion of CD11c(+) dendritic cells abrogates priming of CD8(+) T cells by exogenous cell-associated antigens.. Immunity.

[25] van Rooijen N, Sanders A, van den Berg TK (1996). Apoptosis of macrophages induced by liposome-mediated intracellular delivery of clodronate and propamidine.. J Immunol Methods.

[26] Asselin-Paturel C, Brizard G, Pin JJ, Briere F, Trinchieri G (2003). Mouse strain differences in plasmacytoid dendritic cell frequency and function revealed by a novel monoclonal antibody.. J Immunol.

[27] Allan RS, Waithman J, Bedoui S, Jones CM, Villadangos JA (2006). Migratory dendritic cells transfer antigen to a lymph node-resident dendritic cell population for efficient CTL priming.. Immunity.

[28] Chen J, Trounstine M, Alt FW, Young F, Kurahara C (1993). Immunoglobulin gene rearrangement in B cell deficient mice generated by targeted deletion of the JH locus.. Int Immunol.

[29] Nussler A, Follezou JY, Miltgen F, Mazier D (1989). Effect of irradiation on Plasmodium sporozoites depends on the species of hepatocyte infected.. Trop Med Parasitol.

[30] Silvie O, Semblat JP, Franetich JF, Hannoun L, Eling W (2002). Effects of irradiation on Plasmodium falciparum sporozoite hepatic development: implications for the design of pre-erythrocytic malaria vaccines.. Parasite Immunol.

[31] Sykulev Y, Joo M, Vturina I, Tsomides TJ, Eisen HN (1996). Evidence that a single peptide-MHC complex on a target cell can elicit a cytolytic T cell response.. Immunity.

[32] Berenzon D, Schwenk RJ, Letellier L, Guebre-Xabier M, Williams J (2003). Protracted protection to Plasmodium berghei malaria is linked to functionally and phenotypically heterogeneous liver memory CD8+ T cells.. J Immunol.

[33] Mueller AK, Deckert M, Heiss K, Goetz K, Matuschewski K (2007). Genetically attenuated Plasmodium berghei liver stages persist and elicit sterile protection primarily via CD8 T cells.. Am J Pathol.

[34] Belnoue E, Costa FT, Frankenberg T, Vigario AM, Voza T (2004). Protective T cell immunity against malaria liver stage after vaccination with live sporozoites under chloroquine treatment.. J Immunol.

[35] Hafalla JC, Sano G, Carvalho LH, Morrot A, Zavala F (2002). Short-term antigen presentation and single clonal burst limit the magnitude of the CD8(+) T cell responses to malaria liver stages.. Proc Natl Acad Sci U S A.

[36] Leiriao P, Mota MM, Rodriguez A (2006). Reply to Rénia et al.. The Journal of Infectious Diseases.

[37] Putrianti ED, Silvie O, Kordes M, Borrmann S, Matuschewski K (2009). Vaccine-like immunity against malaria by repeated causal-prophylactic treatment of liver-stage Plasmodium parasites.. J Infect Dis.

[38] Taylor WR, White NJ (2004). Antimalarial drug toxicity: a review.. Drug Saf.

[39] Romero P, Maryanski JL, Corradin G, Nussenzweig RS, Nussenzweig V (1989). Cloned cytotoxic T cells recognize an epitope in the circumsporozoite protein and protect against malaria.. Nature.

[40] Hafalla JC, Rai U, Morrot A, Bernal-Rubio D, Zavala F (2006). Priming of CD8+ T cell responses following immunization with heat-killed Plasmodium sporozoites.. Eur J Immunol.

[41] Hay SI, Rogers DJ, Toomer JF, Snow RW (2000). Annual Plasmodium falciparum entomological inoculation rates (EIR) across Africa: literature survey, Internet access and review.. Trans R Soc Trop Med Hyg.

[42] Bilsborough J, Carlisle M, Good MF (1993). Identification of Caucasian CD4 T cell epitopes on the circumsporozoite protein of Plasmodium vivax. T cell memory.. J Immunol.

[43] Zevering Y, Khamboonruang C, Rungruengthanakit K, Tungviboonchai L, Ruengpipattanapan J (1994). Life-spans of human T-cell responses to determinants from the circumsporozoite proteins of Plasmodium falciparum and Plasmodium vivax.. Proc Natl Acad Sci U S A.

[44] Doolan DL, Hoffman SL, Southwood S, Wentworth PA, Sidney J (1997). Degenerate cytotoxic T cell epitopes from P. falciparum restricted by multiple HLA-A and HLA-B supertype alleles.. Immunity.

[45] Cockburn IA, Chakravarty S, Overstreet MG, Garcia-Sastre A, Zavala F (2008). Memory CD8+ T cell responses expand when antigen presentation overcomes T cell self-regulation.. J Immunol.

[46] Khanna KM, Aguila CC, Redman JM, Suarez-Ramirez JE, Lefrancois L (2008). In situ imaging reveals different responses by naive and memory CD8 T cells to late antigen presentation by lymph node DC after influenza virus infection.. Eur J Immunol.

[47] Probst HC, Tschannen K, Odermatt B, Schwendener R, Zinkernagel RM (2005). Histological analysis of CD11c-DTR/GFP mice after in vivo depletion of dendritic cells.. Clin Exp Immunol.

[48] Belz GT, Smith CM, Kleinert L, Reading P, Brooks A (2004). Distinct migrating and nonmigrating dendritic cell populations are involved in MHC class I-restricted antigen presentation after lung infection with virus.. Proc Natl Acad Sci U S A.

[49] Inaba K, Turley S, Yamaide F, Iyoda T, Mahnke K (1998). Efficient presentation of phagocytosed cellular fragments on the major histocompatibility complex class II products of dendritic cells.. J Exp Med.

[50] Lee JW, Epardaud M, Sun J, Becker JE, Cheng AC (2007). Peripheral antigen display by lymph node stroma promotes T cell tolerance to intestinal self.. Nat Immunol.

[51] Gonzalez M, Mackay F, Browning JL, Kosco-Vilbois MH, Noelle RJ (1998). The sequential role of lymphotoxin and B cells in the development of splenic follicles.. J Exp Med.

[52] Fu YX, Huang G, Wang Y, Chaplin DD (1998). B lymphocytes induce the formation of follicular dendritic cell clusters in a lymphotoxin alpha-dependent fashion.. J Exp Med.

[53] Kamath AT, Henri S, Battye F, Tough DF, Shortman K (2002). Developmental kinetics and lifespan of dendritic cells in mouse lymphoid organs.. Blood.

[54] Sano G, Hafalla JC, Morrot A, Abe R, Lafaille JJ (2001). Swift development of protective effector functions in naive CD8(+) T cells against malaria liver stages.. J Exp Med.

[55] Bruna-Romero O, Hafalla JC, Gonzalez-Aseguinolaza G, Sano G, Tsuji M (2001). Detection of malaria liver-stages in mice infected through the bite of a single Anopheles mosquito using a highly sensitive real-time PCR.. Int J Parasitol.

[56] Zammit DJ, Cauley LS, Pham QM, Lefrancois L (2005). Dendritic cells maximize the memory CD8 T cell response to infection.. Immunity.

[57] Masopust D, Vezys V, Marzo AL, Lefrancois L (2001). Preferential localization of effector memory cells in nonlymphoid tissue.. Science.

[58] Barnes E, Ward SM, Kasprowicz VO, Dusheiko G, Klenerman P (2004). Ultra-sensitive class I tetramer analysis reveals previously undetectable populations of antiviral CD8+ T cells.. Eur J Immunol.

[59] Obar JJ, Khanna KM, Lefrancois L (2008). Endogenous naive CD8+ T cell precursor frequency regulates primary and memory responses to infection.. Immunity.

[60] Scriba TJ, Purbhoo M, Day CL, Robinson N, Fidler S (2005). Ultrasensitive detection and phenotyping of CD4+ T cells with optimized HLA class II tetramer staining.. J Immunol.

