# Supplementary figures and images for: Prolonged Antigen Presentation Is Required for Optimal CD8+ T Cell Responses against Malaria Liver Stage Parasites
Source: PLoS Pathog. 2010 May 6;6(5):e1000877. doi: 10.1371/journal.ppat.1000877 (PMC2865532; doi:10.1371/journal.ppat.1000877)

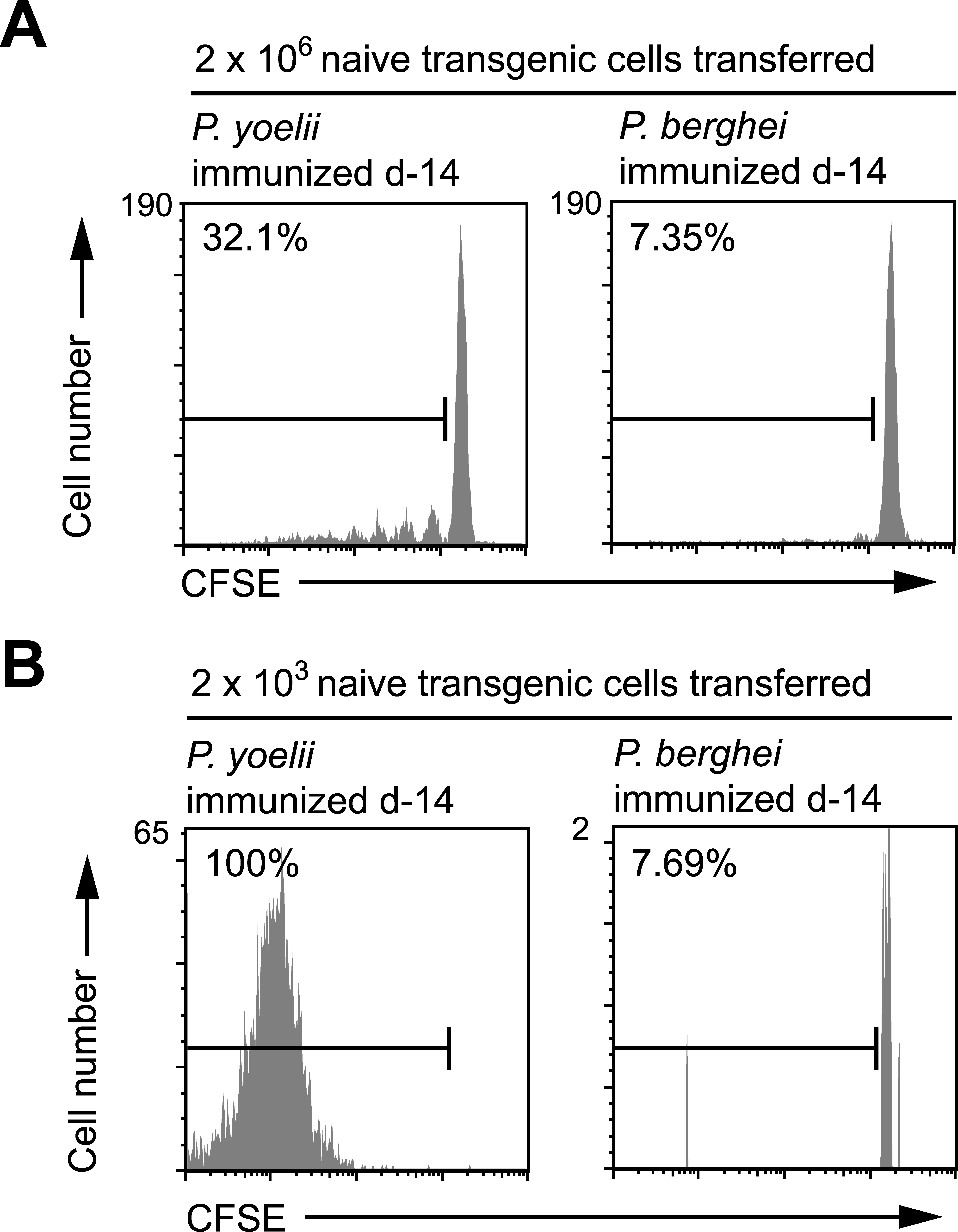

Supplement: Figure S1 — Proliferation of transgenic cells is not due to an ongoing inflammatory response. A. 2 × 10^6^ Thy1.1+ CD8+ T cells from TCR transgenic were transferred into mice immunized 2 weeks previously with either 5 × 10^4^ irradiated P. yoelii sporozoites or 5 × 10^4^ irradiated P. berghei sporozoites. 10 days after cell transfer the spleen cells from the recipient mice were analyzed by FACs. Histograms show representative CFSE profiles of the Thy1.1+ CD8+ cell populations in each group. (n = 3, data from one of two similar experiments shown). B. As in A except only 2 × 10^3^ transgenic cells were transferred. (2.25 MB TIF) [file ppat.1000877.s001.tif]

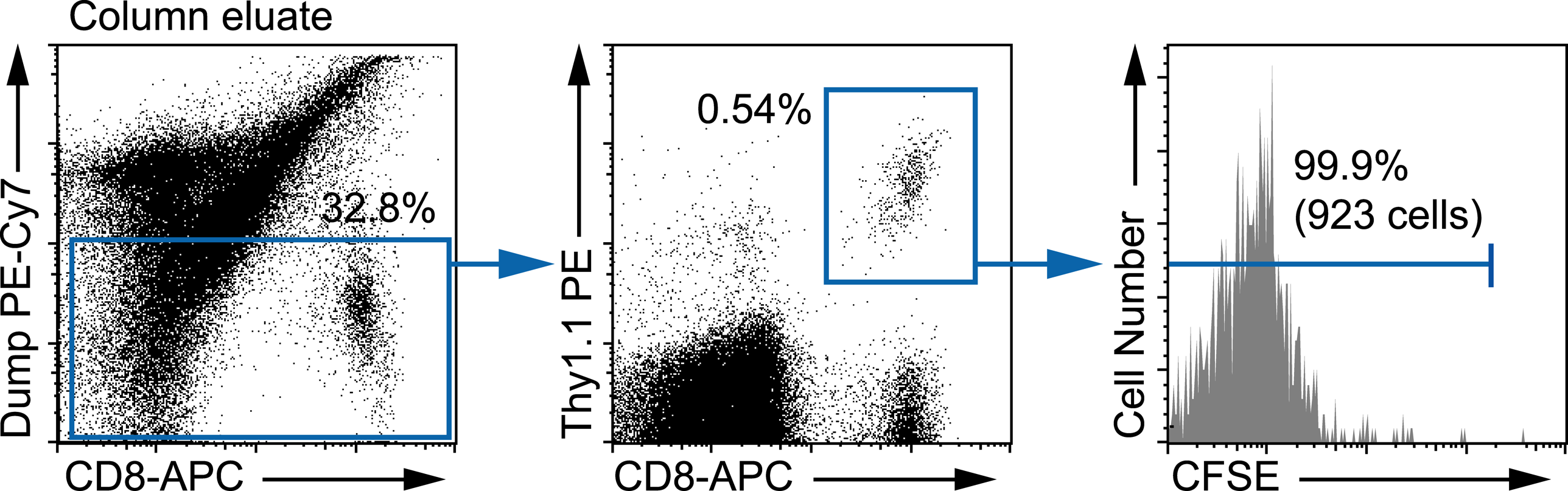

Supplement: Figure S2 — Identification of rare transgenic cell populations. 2 × 10^3^ CFSE labeled transgenic cells were transferred to mice that had been immunized 14 days previously with 5 × 10^4^ irradiated sporozoites. 10 days later the mice were sacrificed and the spleens taken. The cells were stained with anti-Thy1.1 PE and then incubated with anti PE-microbeads (Milteyi Biotech) before positive selection using an LS column. Cells were then counter stained with anti CD11b, anti-CD4 and anti-B220 (all conjugated to PE-Cy7) and anti-CD8 APC and analyzed by FACs. Rare transgenic cells could then be identified using the gating protocol shown and the extent of proliferation determined from CFSE dilution. (1.71 MB TIF) [file ppat.1000877.s002.tif]

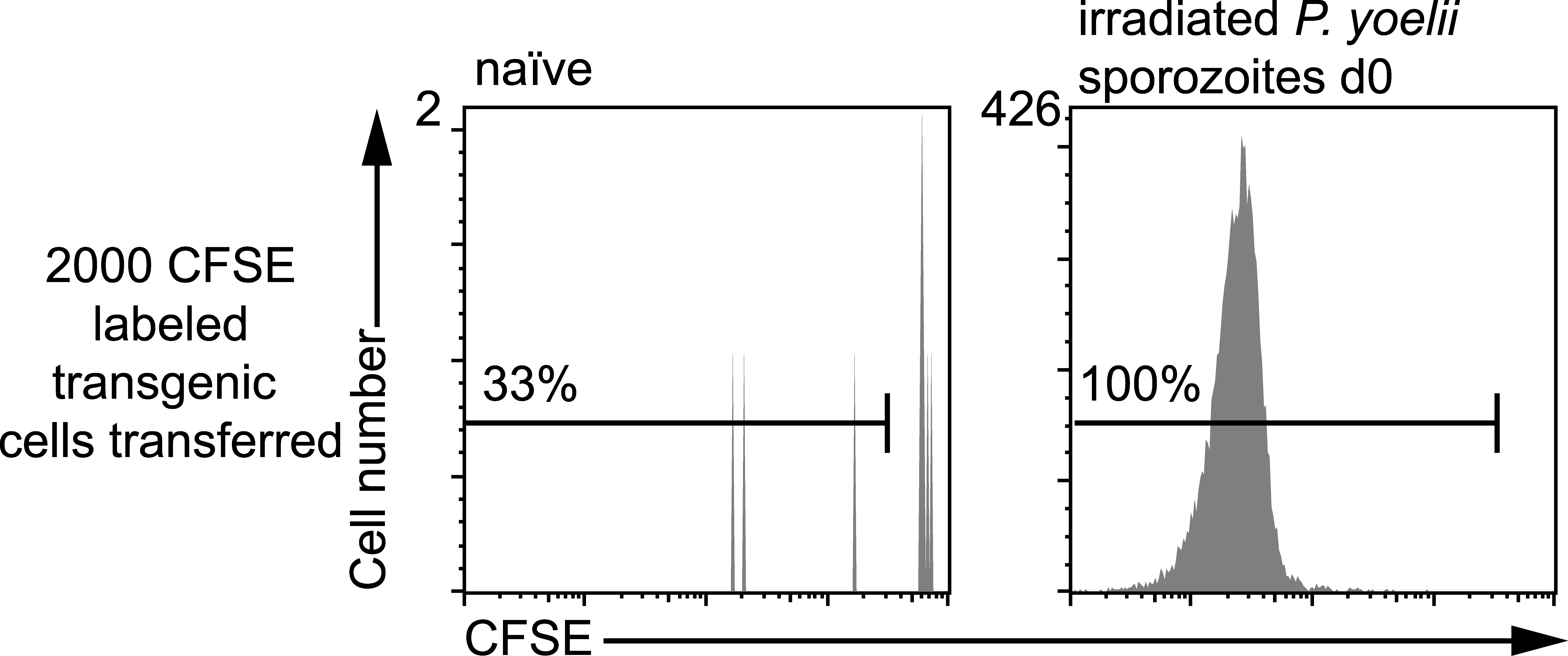

Supplement: Figure S4 — Proliferation of cells four days after immunization. 2 × 10^3^ CFSE labeled Thy1.1 transgenic cells were transferred to mice which were immunized with 5 × 10^4^ P. yoelii irradiated sporozoites. Four days later the Thy1.1+ cells were enriched as described and the proliferation of T cells was assessed by CFSE dilution and compared to naïve controls (representative data from one of three mice per group shown). (1.09 MB TIF) [file ppat.1000877.s004.tif]

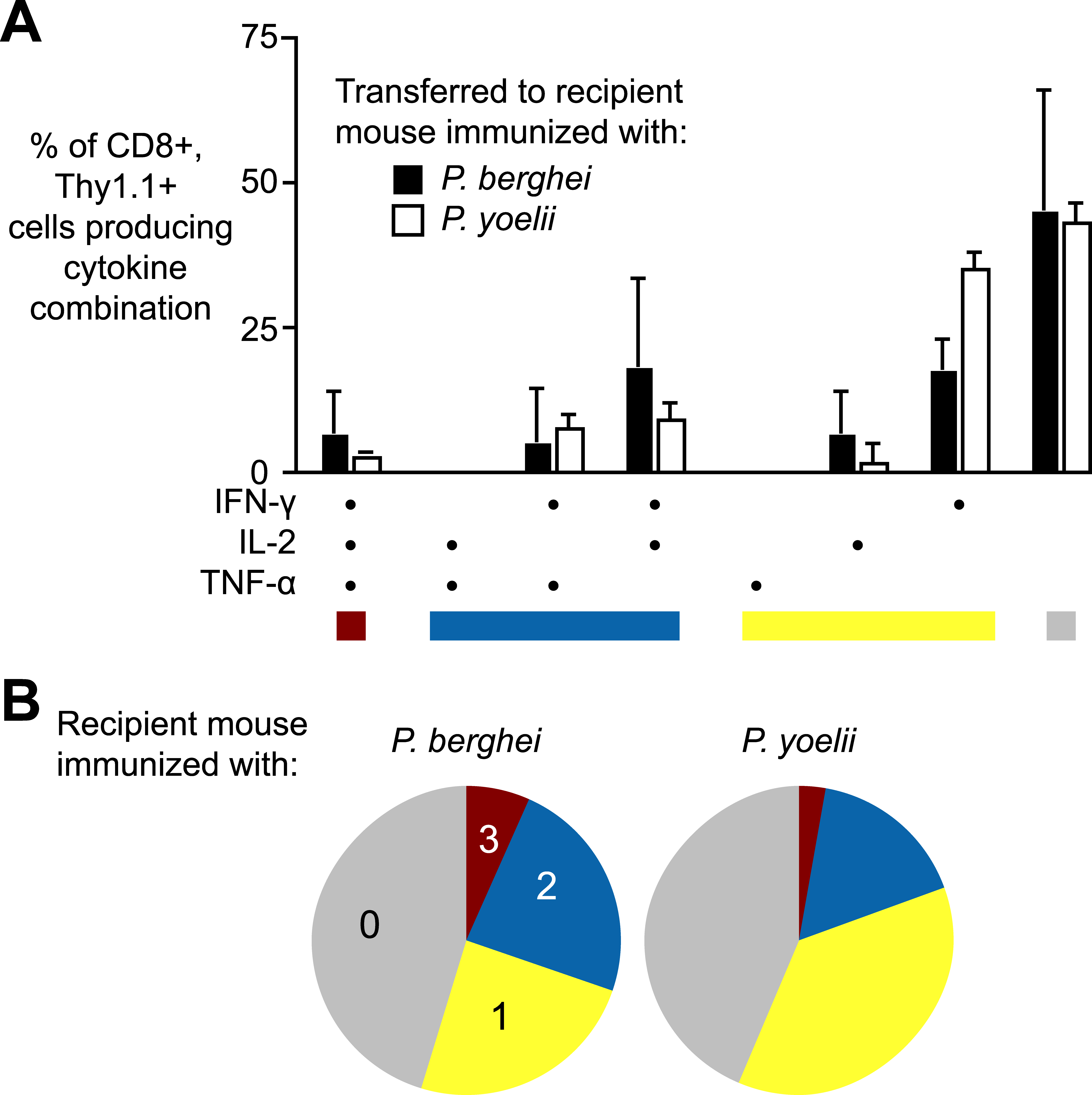

Supplement: Figure S5 — Polyfunctional profiles of cells transferred to P. yoelii and P. berghei immunized mice. Polyfunctional analysis of transgenic T cells in Figure 6Bi: Naïve mice received 2 × 10^3^ transgenic cells and were immunized on d0 with 5 × 10^4^ irradiated P. yoelii sporozoites. On either d4 CD8+ T cells were purified from these mice and 5 × 10^4^ transgenic cells were transferred to recipients that had received 5 × 10^4^ irradiated P. yoelii sporozoites or 5 × 10^4^ irradiated P. berghei sporozoites on d0. On d35 polyfunctional analysis was performed on transgenic T cells recovered from these mice. A. Histograms showing the polyfunctional profile of transgenic cells that had been transferred to P. berghei immunized (black bars) and P. yoelii immunized mice (white bars) 4 days after priming (n = 3, mean ± SE). B. Pie charts showing the number of transgenic cells producing 3, (red), 2 (blue), 1 (yellow) or 0 (gray) cytokines. (3.03 MB TIF) [file ppat.1000877.s005.tif]

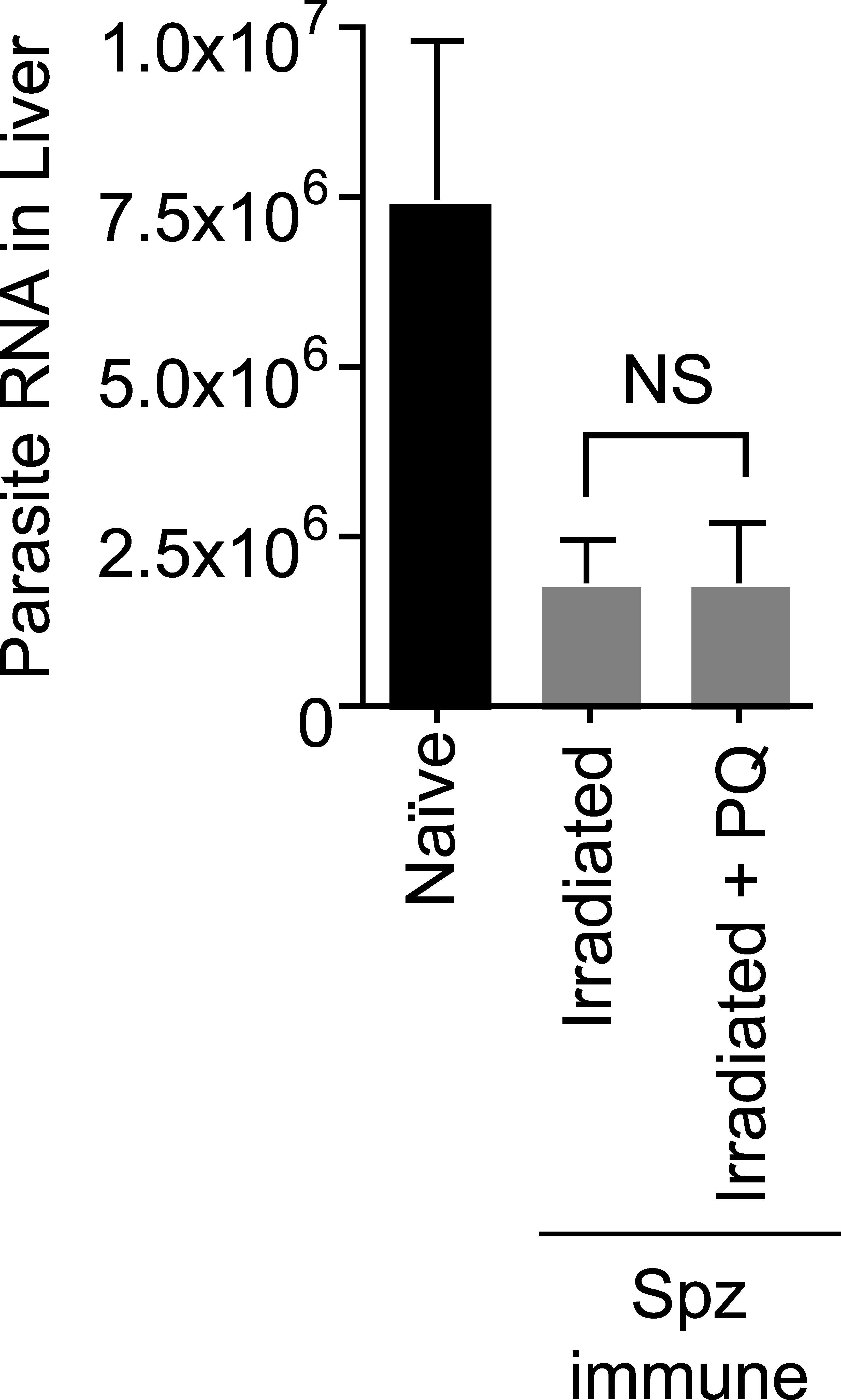

Supplement: Figure S7 — Primaquine treatment does not affect parasite killing in sporozoite immunized mice. Mice were immunized i.v. with 5 × 10^4^ irradiated sporozoites (gray bars) and challenged 37 days later with 5 × 10^3^ live sporozoites with or without primaquine treatment (as in A) on day 7. 40 hours after challenge parasite load was measured in the liver by RT-PCR and compared to naïve controls (black bars; n = 3, mean ± SE). (1.20 MB TIF) [file ppat.1000877.s007.tif]
